# Super responders to biologic therapy in psoriasis: definitions, predictors, and implications for precision medicine

**DOI:** 10.3389/fimmu.2026.1744951

**Published:** 2026-03-13

**Authors:** Xiya Peng, Zhenhua Wang, Kun Han

**Affiliations:** 1 Department of Dermatology and Venerology, Rare Diseases Center, West China Hospital, Sichuan University, Chengdu, China; 2 Department of Dermatology, Weifang People’s Hospital, Weifang, China; 3 Department of Nuclear Medicine, Weifang People’s Hospital, Weifang, China

**Keywords:** psoriasis, biologic, super responder, precision medicine, predictive factor

## Abstract

Biologic therapies have revolutionized psoriasis management, yet treatment responses remain highly heterogeneous. A distinct subgroup of patients, termed super responders (SRs), achieves exceptionally rapid, complete, and sustained responses to biologic therapies. Understanding this phenotype is critical for advancing precision medicine in psoriasis. This review summarizes the latest advances in SRs, focusing on definitions, predictors, and therapeutic implications. Definitions of SRs vary widely across studies, differing in both temporal criteria and efficacy endpoints. Meanwhile, emerging evidence suggests a convergent trend toward multidimensional definitions that combine rapid and complete skin clearance (typically PASI 100 within 3–6 months) and sustained low disease activity during long-term follow-up (often ≥1 year). Convergent predictors of SRs include a lower body mass index (BMI), a favorable metabolic profile, biologic-naïve status and emerging genetic and immunological markers. As a potentially biologically distinct subgroup, SRs present a unique opportunity for treatment optimization, including dosing-interval extension and treatment-free remission in selected patients, offering the potential to sustain efficacy while reducing drug exposure, cost, and patient burden. Key research priorities include establishing consensus definitions, developing validated predictive models, and generating long-term safety data to guide treatment optimization. Integrating the SRs concept into practice may transform psoriasis care from fixed, lifelong regimens to adaptive, evidence-based management grounded in precision medicine.

## 1 Introduction

Psoriasis (PsO) is a prevalent, chronic, inflammatory skin disease that affects over 60 million individuals worldwide, imposing a considerable burden on both patients and society (1). Conventional therapeutic interventions, including topical agents, phototherapy, and systemic immunosuppressants, offer only moderate efficacy, and their long-term utilization is frequently constrained by adverse events or an inadequate response in a proportion of patients (2, 3).

The introduction of biologic therapies in the early 21st century, beginning with tumor necrosis factor-alpha (TNF-α) inhibitors such as adalimumab and infliximab, marked a paradigm shift in the management of psoriasis (4, 5). More recently, the development of biologics targeting specific interleukins (ILs), including IL-17, IL-23, and IL-12/23, has further improved treatment precision and clinical outcomes (6, 7). Despite these advances, treatment responses remain highly heterogeneous. While some patients achieve rapid and complete skin clearance (PASI 100) with sustained remission, others derive limited benefit or experience a gradual loss of efficacy over time (8). This variability highlights a critical unmet need in psoriasis management: identifying distinct responder subgroups, stratifying patients accordingly, and guiding individualized therapeutic strategies.

In this context, the concept of SRs has emerged to describe a distinct subgroup of patients who achieve exceptionally rapid, complete, and sustained responses to biologic therapy (9, 10). Investigating this population holds substantial clinical value, as it may reveal biomarkers and predictive factors associated with optimal efficacy, thereby paving the way for personalized and more effective treatment approaches. This review summarizes current evidence on SRs to biologic therapy in psoriasis, with a focusing on definitions, predictive factors, and potential for optimizing treatment, while also highlighting key directions for future research.

## 2 Definition of super responders

A major barrier to advancing research on SRs is the absence of a standardized, consensus-based definition. Published studies use heterogeneous criteria, with variations in the time points used to assess response and the specific clinical efficacy endpoints required. **Table 1** provides an overview of the diverse definitions of SRs reported in the literature, highlighting the variation in evaluation time points and clinical efficacy endpoints across studies. For completeness, the full textual definitions from individual studies are presented in **Supplementary Table 1**.

**Table 1:** Overview of definitions of SRs to biologic therapy in psoriasis across published studies.

Author	Region	Study	Biologic	Definition of SRs used												
		design		W4	W8	W12	W16	W20	W24	W28	W32	W48	W52	W88	W100	Y5
Mortato et al. (14)	Italy	OBS	Guselkumab					PASI 100								
Marcelli et al. (15)	Italy	OBS	Guselkumab					PASI 100								
Schäkel et al. (10)	Multinational, Europe	RCT	Guselkumab					PASI 100		PASI 100						
Eyerich et al. (12)	Multinational, Europe	RCT	Guselkumab					PASI 100		PASI 100						
Reich et al. (13)	Multinational	RCT^#^	Guselkumab					PASI 100		PASI 100						
Menéndez et al. (27)	Spain	OBS	IL-23 inhibitors				PASI 100		PASI 100							
Ruiz-Villaverde et al. (19)	Spain	OBS	Guselkumab			PASI 100			PASI 100							
Ruiz-Villaverde et al. (20)	Spain	OBS	Guselkumab			PASI 100			PASI 100							
Herranz-Pinto et al. (18)	Italy	OBS	Guselkumab			PASI≤2	PASI≤1 (W16 to W52)									
Feldman et al. (17)	Multinational	RCT^#^	Tildrakizumab							PASI 100						
Morelli et al. (28)	Italy	OBS	Secukinumab											PASI 100	PASI 100	
Mastorino et al. (26)	Italy	OBS	Ixekizumab				PASI 100	PASI 100	PASI 100	PASI 100						
Rompoti et al. (21)	Greece	OBS	Brodalumab			PASI ≤1	PASI ≤1									
Liu et al. (29)	China	OBS	Adalimumab			PASI 100			PASI <1		PASI <1					
Morariu et al. (16)	Romania	OBS	Multiple biologics^*^						PASI 100							
Kim et al. (24)	Korea	OBS	Multiple biologics^*^									PASI 100	PASI 100			
Loft et al. (22)	Denmark	OBS	Multiple biologics^*^						PASI<3 (6 months to 5 years)							
Liu et al. (23)	China	OBS	Multiple biologics^*^	PASI 100	PASI<1 (W8 to W48)											

*PASI* psoriasis area and severity index, *TNF* tumor necrosis factor, *IL* interleukine, *RCT* randomized controlled trial, *OBS* observational study, ^*^TNF-a, IL-17, IL-23 and IL-12/23 inhibitors, ^#^Post hoc analysis of RCT.

### 2.1 Time point

A key parameter in defining SRs is the time point used to assess the response, which remains unstandardized across the literature. This reflects an inherent challenge between capturing a rapid initial response and confirming its long-term durability. The majority of studies, particularly those evaluating a single biologic agent, employ relatively short-term temporal criteria—typically around six months—to define the initial SRs (11–21). For example, in the phase 3b GUIDE trial of guselkumab, patients who achieved complete skin clearance (PASI 100) at weeks 20 and 28 were classified as SRs (10). This benchmark has proven influential and has subsequently been adopted by several subsequent studies (11–13). In contrast, studies involving multiple biologics often use longer-term endpoints, such as 12 months, to minimize potential confounding factors arising from differences in drug mechanisms and onset of action (22–25). Moreover, some studies incorporate not only the initial time point but also the durability of SRs status in order to emphasize sustained efficacy (18, 22, 23, 26). For instance, a retrospective study involving multiple biologics defined SRs as patients who achieved PASI 100 at week 4 and maintained a PASI score below 1 through week 48 (23). Collectively, these findings underscore a growing consensus that an optimal definition of SRs should encompass not only the rapid attainment of clear skin but also the maintenance of that clearance over a clinically meaningful duration.

### 2.2 Clinical efficacy endpoints

Across studies, PASI 100 remains the most widely adopted endpoint, representing complete skin clearance and the highest level of treatment response (11–16, 19, 20, 24, 26–28). As an aspirational benchmark for initial response, it is impractical for long-term assessment in clinical practice because of its stringency. The reason for this is that minor disease fluctuations often occur due to factors such as stress or intercurrent illness. Consequently, absolute PASI thresholds (e.g., PASI ≤ 1) are increasingly recognized as a more pragmatic endpoint for evaluating sustained disease control and have been implemented in several SRs studies, particularly those assessing very early or long-term outcomes (21, 23, 29). This approach aligns with a broader shift toward absolute outcome measures, as reflected in the National Psoriasis Foundation’s (NPF) "Treat-to-Target" recommendations. In addition, patient-reported outcomes (PROs)—such as the Dermatology Life Quality Index (DLQI) and the pruritus Visual Analogue Scale (VAS)—are occasionally incorporated as secondary endpoints (19, 30). While these tools provide a broader evaluation of treatment impact, their inherent subjectivity requires careful interpretation.

### 2.3 Class-specific differences in super-responder definitions between IL-17 and IL-23 inhibitors

Building on the heterogeneity in time points and efficacy endpoints discussed above, emerging differences can be observed in how SRs are operationally defined across biologic classes, particularly between IL-17 inhibitors and IL-23 inhibitors. These differences primarily reflect distinct response kinetics and study design considerations rather than fundamentally different conceptualizations of SRs.

IL-17 inhibition is typically associated with a rapid onset of clinical improvement, consistent with its downstream position in the inflammatory cascade and direct effects on keratinocyte-driven inflammation (7). Accordingly, SRs in IL-17 inhibitors studies are commonly defined using early assessment milestones, including early achievement of PASI ≤1 or PASI 100 (e.g., within 12 weeks) or repeated PASI 100 responses across early visits (21, 26). In contrast, IL-23 inhibition targets an upstream regulatory cytokine that sustains the Th17 program, and its clinical effects tend to emerge more gradually (8). This delayed response has been attributed to progressive modulation of pathogenic Th17 activity and, in some studies, reductions in skin-resident memory T cells (8). Reflecting this response trajectory, SRs definitions in IL-23 inhibitors studies more often rely on later assessment time points, frequently around 20–28 weeks, with PASI 100 serving as the primary efficacy benchmark in alignment with dosing intervals (11–13).

Despite these temporal differences, a convergent trend is increasingly apparent across both therapeutic classes. Recent studies, particularly those evaluating multiple biologics including both IL-17 and IL-23 inhibitors, have moved beyond reliance on a single time-point PASI 100 and instead emphasize definitions that integrate rapid attainment of near-complete or complete skin clearance with sustained disease control over time (18, 22, 23). In this context, absolute PASI thresholds, such as PASI ≤1, are increasingly adopted to characterize sustained disease control, consistent with modern treat-to-target principles and well suited to real-world clinical practice (31).

### 2.4 Emerging trends in defining super responders

Taken together, existing studies indicate an emerging convergence in how SRs to biologic therapy in psoriasis are defined, despite some remaining methodological heterogeneity. Rather than relying on a single parameter, contemporary definitions increasingly adopt a multidimensional framework that integrates the speed and depth of initial response with the durability of therapeutic benefit over time. Specifically, SRs definitions commonly emphasize an early assessment window, typically within 3–6 months, to capture rapid and exceptional treatment responses while accommodating differences in onset kinetics across biologic classes. At the same time, there is a clear shift away from definitions based solely on a single early time point toward approaches that incorporate response durability, reflecting the recognition that sustained disease control is a defining feature of true SRs. Finally, although complete skin clearance (PASI 100) remains the predominant marker of exceptional early efficacy, longer-term evaluation increasingly relies on absolute PASI thresholds, such as PASI ≤1, to represent practical maintenance targets aligned with treat-to-target principles. Nevertheless, the absence of a unified, consensus-based definition continues to limit cross-study comparability and the translation of SRs research into clinical decision-making. Addressing this gap will require the development and validation of harmonized SRs frameworks through prospective, multicenter studies and consensus-driven approaches.

## 3 Predictive factors for super responders

**Figure 1:**
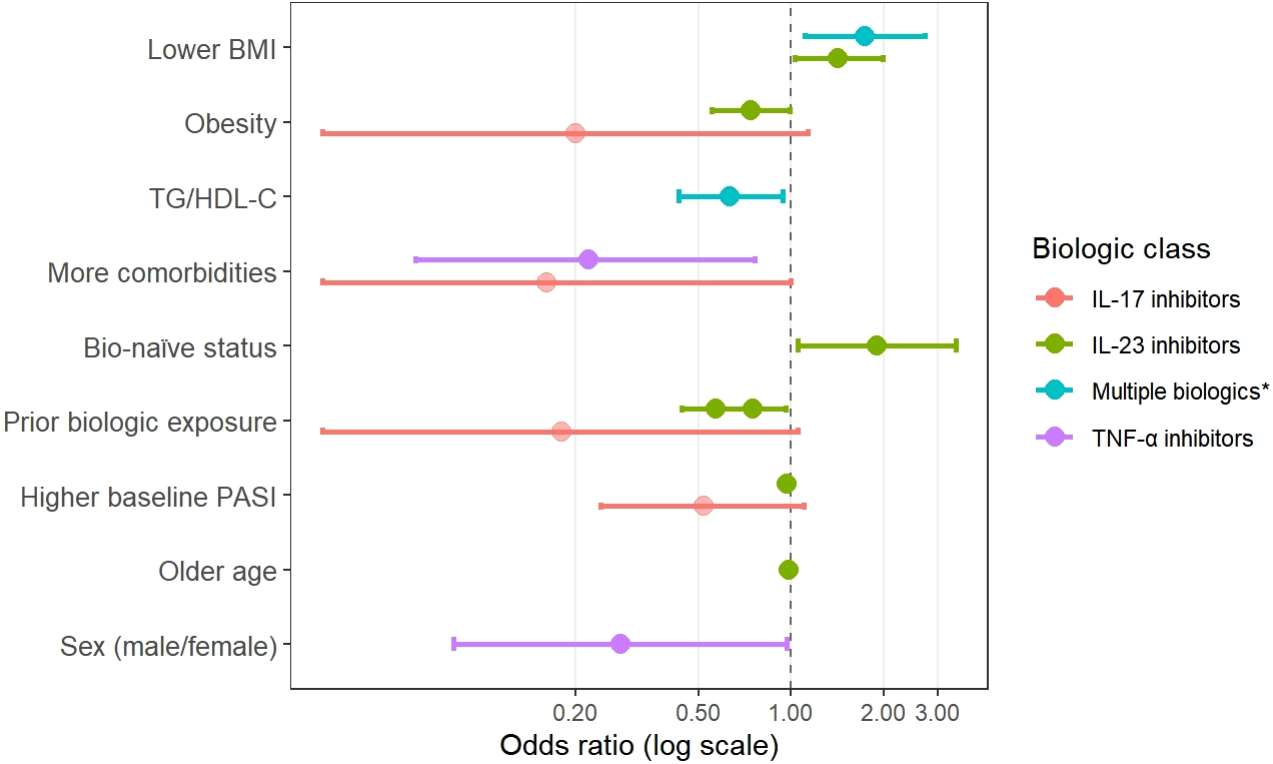
Predictive factors associated with super response to biologic therapy in psoriasis. Dots represent odds ratios (ORs); horizontal bars indicate 95% confidence intervals; the dashed line denotes OR = 1. *TNF-a, IL-17, IL-23 and IL-12/23 inhibitors.

Predicting which patients will become SRs is clinically valuable, as it can guide the first-line treatment selection, improve patient counseling and optimize treatment strategies. Although no definitive predictive model currently exists, research has identified several potential predictors across demographic, clinical, and molecular domains. This section summarizes the existing evidence on key factors, including BMI, metabolic comorbidities, prior treatment exposure, baseline disease severity and emerging pharmacogenomic and immunological markers. Key predictive factors associated with SRs to biologic therapy are illustrated in **Figure 1**, while detailed quantitative data (odds ratios and 95% confidence intervals) are provided in **Supplementary Table 2**.

### 3.1 BMI and obesity

BMI is a well-established predictor of response to biologic therapy in psoriasis. Lower BMI is consistently associated with an increased likelihood of SRs across various biologic classes, whereas obesity is linked to diminished efficacy (13, 21, 23, 32, 33). This relationship could be partly explained by pharmacokinetics, as lower BMI can lead to higher serum drug concentrations and improved bioavailability. For example, Reich et al. reported that patients who experienced SRs to guselkumab had significantly higher median serum drug concentrations at week 28 than those who did not, which partly attributed to their lower baseline body weight (mean BMI 27.8 *vs*. 29.1, respectively) (13). A retrospective multi-biologic study further quantified this correlation, which found that a BMI <25 kg/m² was significantly associated with SRs (OR 1.74, 95% CI 1.11–2.71, p<0.05), while a BMI ≥ 30 kg/m² was an independent predictor of being refractory to biologic treatment (OR 4.07, 95% CI 1.68–9.86, p < 0.01) (23). Beyond initial efficacy, elevated BMI has also been associated with reduced drug survival and higher discontinuation rates, further highlighting its broad impact on long-term treatment outcomes (34–36).

Based on these evidence, lifestyle interventions aimed at weight reduction have been shown to improve treatment outcomes in psoriasis (37, 38). In a randomized controlled trial of patients receiving anti-TNF-α therapy, those in the low-calorie diet group achieved a mean weight loss of 12.9 kg by week 24, with 85.9% achieving PASI 75 compared to 59.3% in the control group (p<0.05) (39). Collectively, these results make BMI not only as a key predictive biomarker but also as a modifiable therapeutic target for enhancing biologic efficacy and achieving long-term disease control.

### 3.2 Metabolic profile and comorbidities

Beyond BMI and obesity, a patient’s underlying metabolic profile is emerging as a critical determinant of therapeutic response, with dyslipidemia and insulin resistance being particularly influential (40–42). There is a robust evidence linking dyslipidemia directly to treatment outcomes. A real-world cohort study of patients treated with adalimumab found that higher high-density lipoprotein (HDL) levels were independently associated with PASI 90 response at weeks 24 and 52 (p<0.05), whereas low HDL levels markedly reduced the likelihood of achieving this outcome by 76% at week 24 (OR = 0.24, 95% CI: 0.07–0.79) and 87% at week 52 (OR = 0.13, 95% CI: 0.04–0.44) (29). Furthermore, multiple indicators of insulin resistance have been associated with SRs. SRs tend to exhibit more favorable metabolic characteristics, including lower triglyceride-to-HDL cholesterol (TG/HDL-C) ratios (23) and a lower prevalence of diabetes mellitus than non-SRs (7.4% *vs*. 13.8%, p=0.008) (33). Conversely, patients who are refractory to biologics more frequently present with conditions such as non-alcoholic fatty liver disease (NAFLD), which is strongly linked to insulin resistance (23, 43). Taken together, these findings suggest that metabolic health is an active modulator of biologic efficacy, not merely a comorbidity. Therefore, optimizing a patient’s metabolic profile may therefore be a crucial strategy for enhancing treatment success.

### 3.3 Prior biologic exposure

Prior exposure to biologic agents is a well-studied factor that influences subsequent therapeutic outcomes. Multiple studies have shown that biologic-naïve patients are significantly more likely to become SRs than those previously treated with biologics (20, 32, 42). For example, a retrospective study of guselkumab revealed a significantly higher proportion of biologic-naïve individuals among SRs than among non-SRs (42.4% *vs*. 32.6%; p = 0.002) (32). This finding is further supported by multivariate analyses from another study, which identified biologic-naïve status as an independent predictor of SRs (20).

In contrast, patients with prior exposure to biologics, particularly to multiple agents, tend to exhibit reduced treatment responses and shorter drug survival (29, 32). In a real-world adalimumab cohort, previous biologic exposure was an independent negative predictor of PASI 90 at week 52 (OR = 0.08, 95% CI: 0.01–0.81; p < 0.05) (29). This effect appears to be cumulative: another study reported that patients exposed to two or more prior biologics had the lowest three-year drug survival rates when treated with secukinumab (36). Taken together, these findings emphasize the importance of optimizing the initial selection of biologics to maximize the potential for an exceptional and durable long-term response.

### 3.4 Baseline disease severity

The role of baseline disease severity as a predictor of SRs remains controversial. It has been reported by some studies that lower initial disease activity is associated with more favorable outcomes. For instance, a large retrospective study of guselkumab reported that SRs had a significantly lower mean baseline PASI score compared to non-SRs (13.4 *vs*. 15.5; p = 0.001) (14). Similarly, pooled analyses of the VOYAGE-1 and VOYAGE-2 trials demonstrated that SRs were more likely to achieve early responses, such as PASI 75 at week 2 (5.5% *vs*. 1.8%) and PASI 100 at week 8 (22.5% *vs*. 3.3%), compared with non-SRs (13). However, contradictory evidence exists. A retrospective analysis of patients treated with IL-17 and IL-23 inhibitors found that SRs presented with significantly higher initial PASI scores than non–SRs (15.3 *vs*. 14.4; p = 0.037) (33). These divergent findings likely reflect differences in study populations and unmeasured confounding factors, rather than a true causal effect of baseline PASI alone. In particular, variability in patient characteristics such as body mass index, which can influence treatment response and drug exposure, as well as differences in the biologic classes included across studies, may modify or obscure the relationship between baseline disease severity and SRs. This inconsistency suggests that baseline PASI may not be a straightforward predictor of response, and its predictive value requires clarification in future studies that adopt standardized definitions.

### 3.5 Pharmacogenomic predictors

Pharmacogenomic studies have begun to identify specific genetic markers that may predict SRs to certain biologic therapies (44–46). A pharmacogenetic study of secukinumab analyzed 417 single-nucleotide polymorphisms (SNPs) in 62 psoriasis patients and identified eight SNPs in the HLA-C region and three in other immune-related genes associated with outstanding therapeutic outcomes (28). Notably, the presence of rs34085293 or rs2304255 was found to define a distinct subgroup of SRs of secukinumab. It has been observed that analogous associations between specific drugs and other biologics have been identified. In a cohort of 164 patients treated with ustekinumab, Talamonti et al. found that individuals carrying both the HLA-C06:02 and HLA-C04 alleles represented a clear SRs subgroup, with over 70% achieving complete and sustained remission for up to two years (32).

However, the available pharmacogenomic evidence is still limited. Most studies are exploratory and rely on relatively small cohorts, and independent replication remains scarce (28). In addition, the majority of reported data originate from populations of European ancestry, which may limit applicability to other ethnic groups (28, 32). In line with this, large real-world registries, including BADBIR, have not consistently identified genetic predictors when biologic therapies are assessed collectively (47). Therefore, pharmacogenomic predictors of SRs should be interpreted with caution and require validation in larger, ethnically diverse cohorts.

### 3.6 Emerging immunological predictors

In addition to demographic and clinical characteristics, recent studies have begun to explore the immunological mechanisms underlying SRs, thus offering new opportunities for the discovery of predictive biomarkers. Growing evidence indicates that SRs is not a random phenomenon but reflects a distinct and favorable immunobiological state.

In biologic-naïve patients treated with the IL-17A inhibitor secukinumab, SRs demonstrated a unique systemic cytokine profile even before treatment initiation. Specifically, SRs exhibited elevated baseline IL-13 and reduced IL-18 levels compared with non-SRs, with the IL-18/IL-13 ratio proving to be a strong predictor of SRs (48). These findings suggest that a baseline immune milieu skewed toward Th2 polarization may predispose certain patients to an exceptional response to IL-17A blockade. Distinctive immunological features have also been observed at the tissue level. The persistence of pathogenic tissue-resident memory T (TRM) cells in psoriatic skin is a well-recognized driver of disease recurrence (49–51). In patients receiving the IL-23 inhibitor guselkumab, SRs exhibited a rapid and sustained depletion of pathogenic CD8^+^ TRM within psoriatic lesions, with a corresponding achievement of normalization by week 28 compared with week 68 among non-SRs (26, 52). Notably, this suppression was observed to persist even with extended dosing intervals, suggesting a durable immune resetting within the lesional skin.

Importantly, this favorable immunologic profile appears to represent an intrinsic patient trait rather than a drug-specific effect. In a real-world cohort of patients treated with the TNF-α inhibitor adalimumab, all SRs who subsequently switched to an IL-17A inhibitor achieved PASI 100 by week 4, whereas only 35–43% of non-SRs reached the same response by week 12 (29). This sustained efficacy across different biologic classes supports the concept of SRs as a distinct subgroup characterized by a stable and adaptable immunologic profile.

## 4 Treatment optimization in super responders

Optimization of treatment is a central goal in the management of chronic inflammatory diseases. This process aims to balance sustained efficacy with minimized drug exposure to reduce long-term risks and healthcare costs (53, 54). Such strategies are well established in conditions like rheumatoid arthritis (55, 56), but their application in psoriasis has been comparatively limited, largely due to the absence of robust patient stratification methods. The identification of SRs provides a unique opportunity to address this gap. Given the exceptionally rapid, complete, and durable response of SRs to biologic therapy, it is therefore considered to be an ideal population in which to evaluate treatment optimization strategies, such as dosing interval extension and treatment-free remission.

### 4.1 Dosing interval extension

Given the subcutaneous administration of most biologics, the most extensively investigated optimization approach among SRs has been the extension of the dosing interval. The phase IIIb GUIDE trial provides the first randomized controlled evidence supporting this strategy. In this study, patients treated with guselkumab who achieved SRs status(defined as achieving PASI 100 at both weeks 20 and 28) were randomized to either standard 8-week dosing or an extended 16-week interval (12). By week 68, the extended-interval group demonstrated non-inferior efficacy, maintaining comparable disease control (PASI < 3) to the standard-dosing group. These findings demonstrate that it is possible to extend dosing intervals safely in appropriately selected SRs without compromising clinical outcomes.

Beyond fixed-interval extensions, flexible and individualized strategies have also shown promise. A retrospective study of guselkumab assessed an on-demand strategy in which SRs were retreated only upon clinical relapse (PASI ≥1). Over a 90-week follow-up, this approach reduced the total number of doses by 47% compared with standard protocols, while successfully maintaining therapeutic efficacy (18).

### 4.2 Treatment-free remission

Despite the ongoing debate surrounding the concept of treatment-free remission (TFR) and disease modification in psoriasis, emerging evidence suggests that certain SRs have the capacity to maintain clear or nearly clear skin for extended periods after treatment cessation (57).

The probability of attaining TFR appears to be closely associated with the depth of the initial treatment response. For instance, a comparative analysis of phase III trials for anti-IL-23 inhibitors found that patients who had achieved PASI 90 or better at the time of treatment discontinuation were significantly more likely to remain in remission one year later (58). This finding supports the hypothesis that profound skin clearance—a defining characteristic of SRs—may indicate a biological predisposition toward sustained disease control even after therapy withdrawal.

The principle is further substantiated by clinical evidence. Schäkel et al. reported that SRs were more frequently observed among patients with short disease duration (SDD) (59). Following treatment withdrawal, these SDD patients maintained disease control for a median treatment-free period exceeding one year. Similarly, a nationwide cohort study found that among psoriasis patients who discontinued biologics after achieving complete remission, 43% remained free of systemic therapy for up to two years, requiring at most topical treatments (60).

At present, TFR is not recommended as a routine goal in major international guidelines due to the high risk of relapse in unselected patient populations (61). Nevertheless, the identification of SRs raises the possibility that sustained TFR—at least for a subset of patients—may be an attainable and clinically meaningful objective when supported by appropriate patient selection and monitoring strategies.

## 5 Super responders in psoriasis management: paradigm shift and future directions

The concept of the SRs extends far beyond a simple indicator of therapeutic efficacy; it represents a potential paradigm shift in how treatment goals are defined and psoriasis is managed over the long term (62). Whilst current international guidelines are predicated on general factors such as disease severity, comorbidities, and safety (63, 64), they are deficient in precise, individualized strategies and dynamic long-term protocols. The identification of SRs may be pivotal in addressing these gaps. Recognizing this cohort enables clinicians to pursue more ambitious objectives, such as optimizing medication selection, implementing de-escalation protocols without compromising disease control, and transitioning from population-based standardized therapy to individualized, precision-based management (5, 65). These strategies are consistent with the principles of precision medicine, offering the potential to reduce drug exposure, lower treatment costs, and alleviate patient burden (66). Moreover, they may enhance the evidence base for cost-effectiveness and reimbursement policies by demonstrating that certain patients can sustain long-term remission with reduced dosing (67).

Despite its significant clinical potential, the translation of the SRs concept into standard practice faces several critical challenges. Firstly, a standardized definition is lacking. Existing studies utilize a range of heterogeneous endpoints, from short-term responses at week 12 to sustained remission over several years, and apply varying thresholds, from the stringent PASI 100 to the more pragmatic PASI ≤1. This variability severely hampers cross-trial comparisons and meta-analyses. Secondly, there is a necessity for reliable predictive factors. Although parameters such as lower body mass index, favorable metabolic profiles, and biologic-naïve status have been associated with superior outcomes, findings are not always consistent across studies. These discrepancies are likely to reflect variations in study design (randomized controlled trials versus real-world cohorts), population characteristics, and comparator groups (treatment-resistant versus non-SRs patients). In addition to isolated predictors, there is an urgent requirement to develop comprehensive, validated models that can elucidate underlying mechanisms and guide clinical application for SRs. Finally, there is a paucity of evidence to guide the optimization of treatment in SRs. Although strategies such as dosing interval extension and treatment-free remission have shown promise, there is a lack of standardized protocols. Furthermore, long-term safety outcomes, including relapse risk, rebound phenomena, and retreatment efficacy, also remain insufficiently characterized. The existing data, which is predominantly derived from observational cohorts or *post-hoc* analyses, offers limited generalizability. Large, prospective studies with harmonized definitions and extended follow-up are needed to identify robust predictors and establish evidence-based strategies for optimizing therapy in this distinctive patient subgroup.

## 6 Conclusion

The concept of the SRs marks a pivotal step in the evolution of psoriasis management, signaling a move away from generalized treatment protocols to true precision medicine. This review synthesizes the evidence showing that SRs are not merely at the favorable end of a response spectrum but constitute a distinct subgroup with unique clinical, metabolic, and immunological characteristics that predispose them to exceptional outcomes. Although definitions of SRs have varied across studies, emerging evidence points toward a convergent, multidimensional conceptualization that integrates the speed and depth of initial treatment response with the durability of disease control over time. The ability to prospectively identify SRs using predictive factors such as lower BMI, biologic-naïve status, favorable metabolic profiles will empower clinicians to have more informed discussions with patients about long-term treatment goals. More importantly, it provides a rationale for the implementation of sophisticated management strategies, such as extending dosing interval and treatment-free remission, which were previously been limited by the inability to reliably stratify patients. Future research should integrate multi-omic, clinical, and real-world data to reveal the biological basis of the SRs and to establish durable, patient-centered therapeutic algorithms. Overall, recognizing and studying SRs provides a valuable framework for advancing precision medicine in psoriasis—shifting from fixed, lifelong therapy toward dynamic, evidence-driven, and individualized care that maximizes outcomes while minimizing treatment burden.
