# Supplementary material for: Super responders to biologic therapy in psoriasis: definitions, predictors, and implications for precision medicine
Source: Front Immunol. 2026 Mar 13;17:1744951. doi: 10.3389/fimmu.2026.1744951 (PMC13021661; doi:10.3389/fimmu.2026.1744951)
Supplement: Supplementary file 1 [file Table1.docx]

Supplementary Material

**Supplementary Table S1. Detailed textual definitions of SRs to biologic therapy in psoriasis across published studies.**

| \| Author \| Region \| Study design \| Biologic \| Definition of super responders used \| \| --- \| --- \| --- \| --- \| --- \| | |
| --- | --- | --- | --- | --- | --- | --- |
| \| Mortato et al.^14^ \| Italy \| OBS \| Guselkumab \| Achieving PASI 100 at week 20 of treatment. \| \| --- \| --- \| --- \| --- \| --- \| \| Marcelli et al.^15^ \| Italy \| OBS \| Guselkumab \| Achieving PASI 100 within the first 20 weeks of treatment. \| \| Schäkel et al.^11^ \| Multinational, Europe \| RCT \| Guselkumab \| Achieving PASI = 0 at both weeks 20 and 28 of treatment. \| \| Eyerich et al.^12^ \| Multinational, Europe \| RCT \| Guselkumab \| Achieving PASI = 0 at both weeks 20 and 28 of treatment. \| \| Reich et al.^13^ \| Multinational \| RCT^#^ \| Guselkumab \| Achieving PASI = 0 at both weeks 20 and 28 of treatment. \| \| Menéndez et al.^27^ \| Spain \| OBS \| IL-23 inhibitors \| Achieving PASI 0 at weeks 16 and 24 of treatment. \| \| Ruiz-Villaverde et al.^19^ \| Spain \| OBS \| Guselkumab \| Achieving PASI 0 at weeks 12 and 24 of treatment. \| \| Ruiz-Villaverde et al.^20^ \| Spain \| OBS \| Guselkumab \| Achieving PASI 0 at weeks 12 and 24 of treatment. \| \| Herranz‑Pinto et al.^18^ \| Italy \| OBS \| Guselkumab \| Achieving PASI ≤2 after the third guselkumab dose and maintaining PASI ≤1 for ≥52 weeks. \| \| Feldman et al.^17^ \| Multinational \| RCT^#^ \| Tildrakizumab \| Achieving PASI 90 at week 28 of treatment. \| \| Morelli et al.^28^ \| Italy \| OBS \| Secukinumab \| Maintaining PASI 100 through weeks 88 and 100 of treatment. \| \| Mastorino et al.^26^ \| Italy \| OBS \| Ixekizumab \| Achieving PASI 100 at week 16 and maintaining complete clearance at week 28. \| \| Rompoti et al.^21^ \| Greece \| OBS \| Brodalumab \| Achieving absolute PASI ≤ 1 at weeks 12 or 16 of treatment. \| \| Liu et al.^29^ \| China \| OBS \| Adalimumab \| Achieving PASI 100 at week 12 and maintaining PASI <1 at week 24 or 32. \| \| Morariu et al.^16^ \| Romania \| OBS \| Multiple biologics^*^ \| Achieving PASI 100 at the six-month (approximately week 24) visit. \| \| Kim et al.^24^ \| Korea \| OBS \| Multiple biologics^*^ \| Achieving PASI 100 between weeks 48 and 52 of treatment. \| \| Loft et al.^22^ \| Denmark \| OBS \| Multiple biologics^*^ \| Remaining on first biologic for ≥5 years, maintaining PASI <3 between 6 months and 5 years. \| \| Liu et al.^23^ \| China \| OBS \| Multiple biologics^*^ \| Achieving PASI 100 at week 4 and maintaining PASI <1 through week 48. \| \| Mason et al.^25^ \| UK and Ireland \| OBS \| Multiple biologics^*^ \| Receiving continuous first-line biologic monotherapy for ≥5 years (BADBIR registry). \| |  |

*SRs* super responders, *PASI* psoriasis area and severity index, *TNF* tumor necrosis factor, *IL* interleukine, *RCT* randomized controlled trial, *OBS* observational study, ^*^TNF-a, IL-17, IL-23 and IL-12/23 inhibitors, ^#^Post hoc analysis of RCT.

**Supplementary Table S2. Quantitative data on predictive factors for super response to biologic therapy in psoriasis.**

| \| Author \| Region \| Study design \| Biologic \| Predictive Factors (OR, 95% CI) \| \| \| \| \| \| \| \| \| \| --- \| --- \| --- \| --- \| --- \| --- \| --- \| --- \| --- \| --- \| --- \| --- \| --- \| \| Lower BMI \| Obesity \| TG/HDL-C \| More comorbidities \| Bio-naïve status \| Prior biologic exposure \| Higher baseline PASI \| Older age \| Sex (male/female) \| \| |
| --- | --- | --- | --- | --- | --- | --- | --- | --- | --- | --- | --- | --- | --- | --- | --- | --- | --- | --- | --- | --- | --- | --- | --- |
| \| Liu et al.^23^ \| China \| OBS \| Multiple biologics^*^ \| 1.74 (1.11-2.71) \|  \| 0.63 (0.43-0.94) \|  \|  \|  \|  \|  \|  \| \| --- \| --- \| --- \| --- \| --- \| --- \| --- \| --- \| --- \| --- \| --- \| --- \| --- \| \| Reich et al.^13^ \| Multinational \| RCT^#^ \| IL-23 inhibitors \| 1.42 (1.03-1.98) \|  \|  \|  \|  \|  \| 0.97（0.96-0.99） \| 0.98 (0.97-0.99） \|  \| \| Mortato et al.^14^ \| Italy \| OBS \| IL-23 inhibitors \|  \| 0.74（0.55–0.99） \|  \|  \|  \| 0.57 (0.44–0.75） \| 0.97（0.95–0.98） \|  \|  \| \| Marcelli et al.^15^ \| Italy \| OBS \| IL-23 inhibitors \|  \|  \|  \|  \| 1.90 (1.05-3.43) \| 0.75 (0.58–0.96） \|  \|  \|  \| \| Rompoti et al.^21^ \| Greece \| OBS \| IL-17 inhibitors \|  \| 0.20（0.03–1.13） \|  \| 0.16 (0.03-1.00） \|  \| 0.18 (0.03–1.05） \| 0.52（0.24–1.10） \|  \|  \| \| Liu et al.^29^ \| China \| OBS \| TNF-a inhibitors \|  \|  \|  \| 0.22 (0.06-0.76） \|  \|  \|  \|  \| 0.28 (0.08–0.97) \| |

*TNF* tumor necrosis factor, *IL* interleukine, *RCT* randomized controlled trial, *OBS* observational study, ^*^TNF-a, IL-17, IL-23 and IL-12/23 inhibitors, ^#^Post hoc analysis of RCT.

Data are presented as odds ratios (ORs) with 95% confidence intervals (CIs).
